# Supplementary figures and images for: Myelin regulatory factor (MYRF) is a critical early regulator of retinal pigment epithelial development
Source: PLoS Genet. 2025 Apr 15;21(4):e1011670. doi: 10.1371/journal.pgen.1011670 (PMC12052213; doi:10.1371/journal.pgen.1011670)

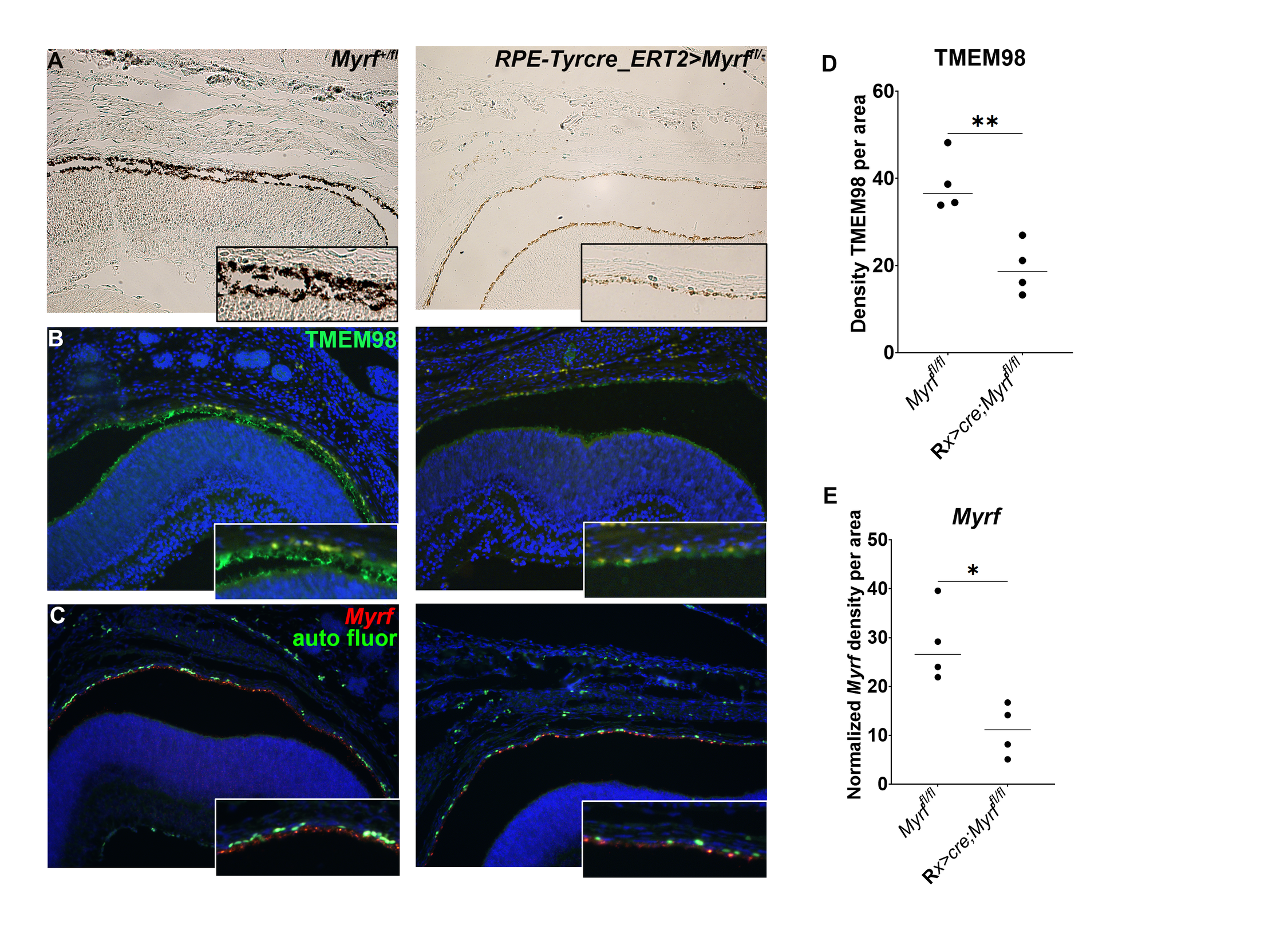

Supplement: S1 Fig — RPE-Tyrcre-ERT2 > Myrffl/- and controls were injected with tamoxifen at e11.5, e12.5, and e13.5, and harvested at e18.5. (A) Pigmentation was reduced in the RPE of RPE-Tyrcre-ERT2 > Myrffl/- mice compared to Myrf+/fl controls. (B) Expression of TMEM98 is reduced in the RPE-Tyrcre-ERT2 > Myrffl/- mice compared to Myrf+/fl controls. (C) Expression of Myrf with RNAscope is reduced in the RPE-Tyrcre-ERT2 > Myrffl/- mice compared to Myrf+/fl controls. The density of fluorescent staining was quantitated in ImageJ for TMEM98 (D) and Myrf (E), normalized to the background staining and expressed as a density per area. Statistics were calculated using the Unpaired T test. (TIF) [file pgen.1011670.s001.tif]

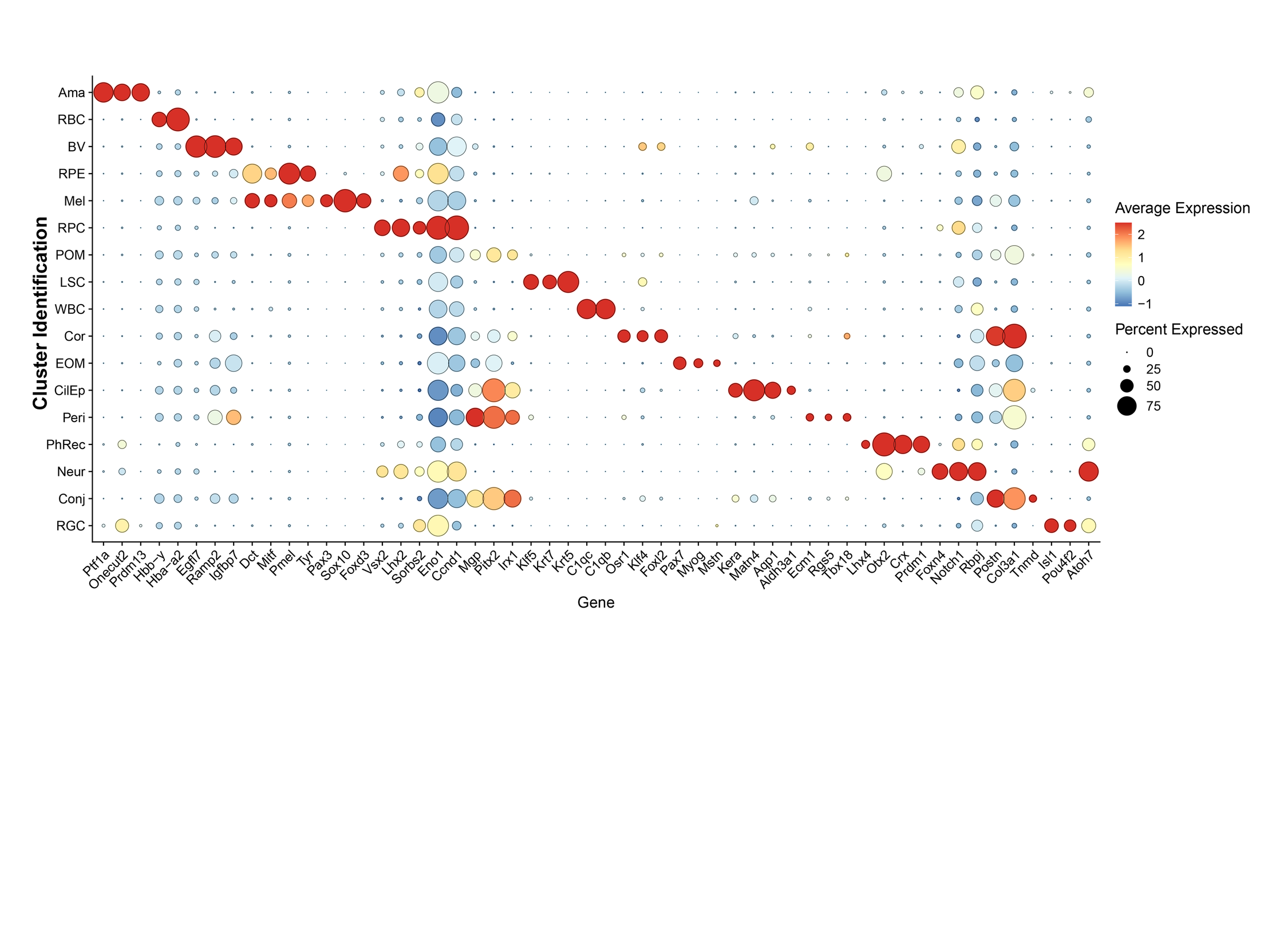

Supplement: S2 Fig — Cluster identification is labeled on the left of the graph and the transcript analyzed is listed at the bottom of the graph. Expression is displayed using a gradient where red signifies high levels of expression and blue indicates low levels of expression. The size of the dot is correlated to the number of cells within a particular cluster that expresses the gene. (TIF) [file pgen.1011670.s002.tif]

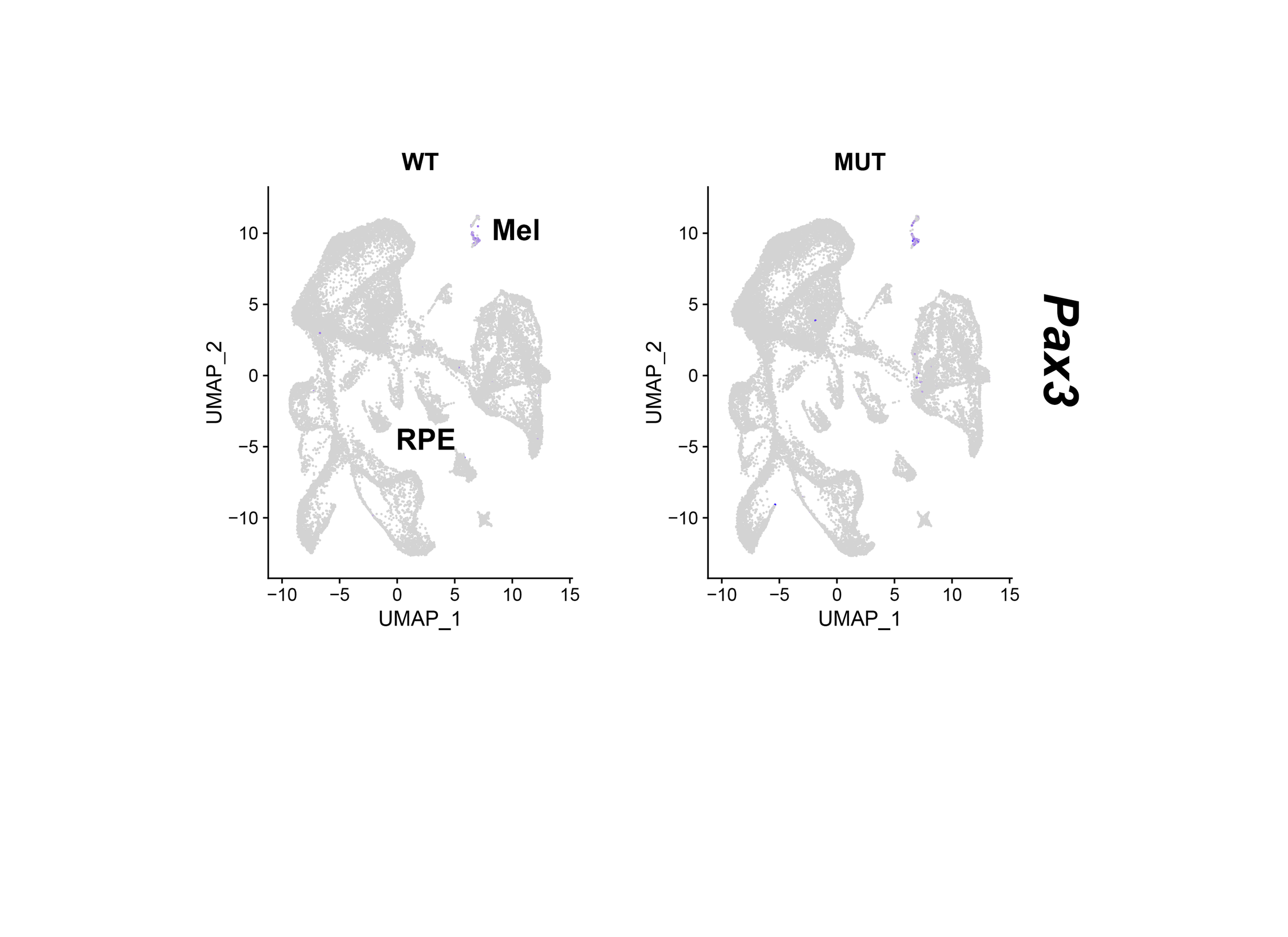

Supplement: S3 Fig — FeaturePlots showing Pax3 is specifically expressed in the melanocyte (Mel) cluster and not the RPE cluster, demonstrating a clear distinction between the two clusters. (TIF) [file pgen.1011670.s003.tif]

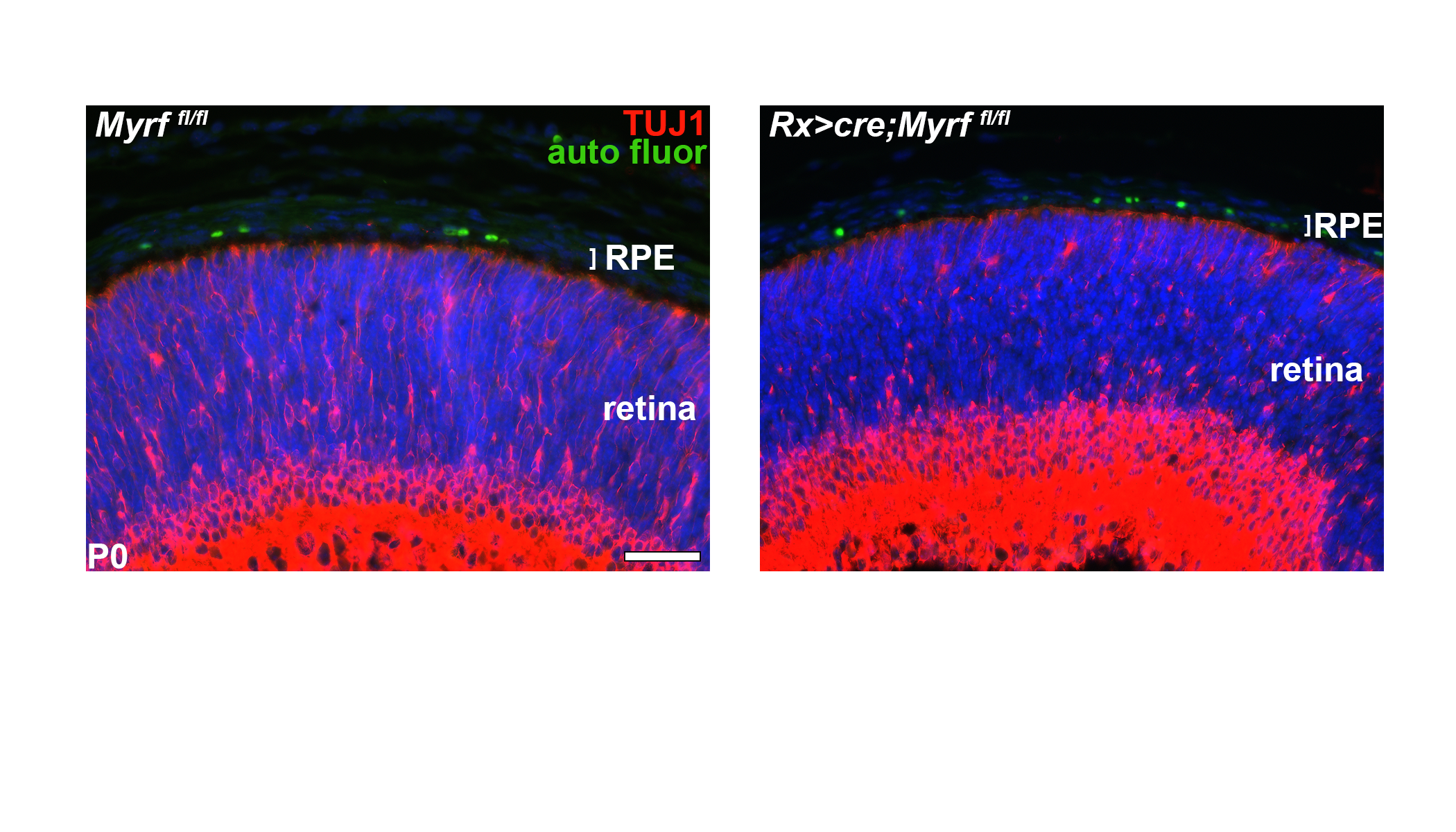

Supplement: S4 Fig — TUJ1 staining is in red and auto fluorescent choroidal red blood cells are seen in green. The RPE and retina layers are labeled for orientation. Scale bar represents 50uM. (TIF) [file pgen.1011670.s004.tif]

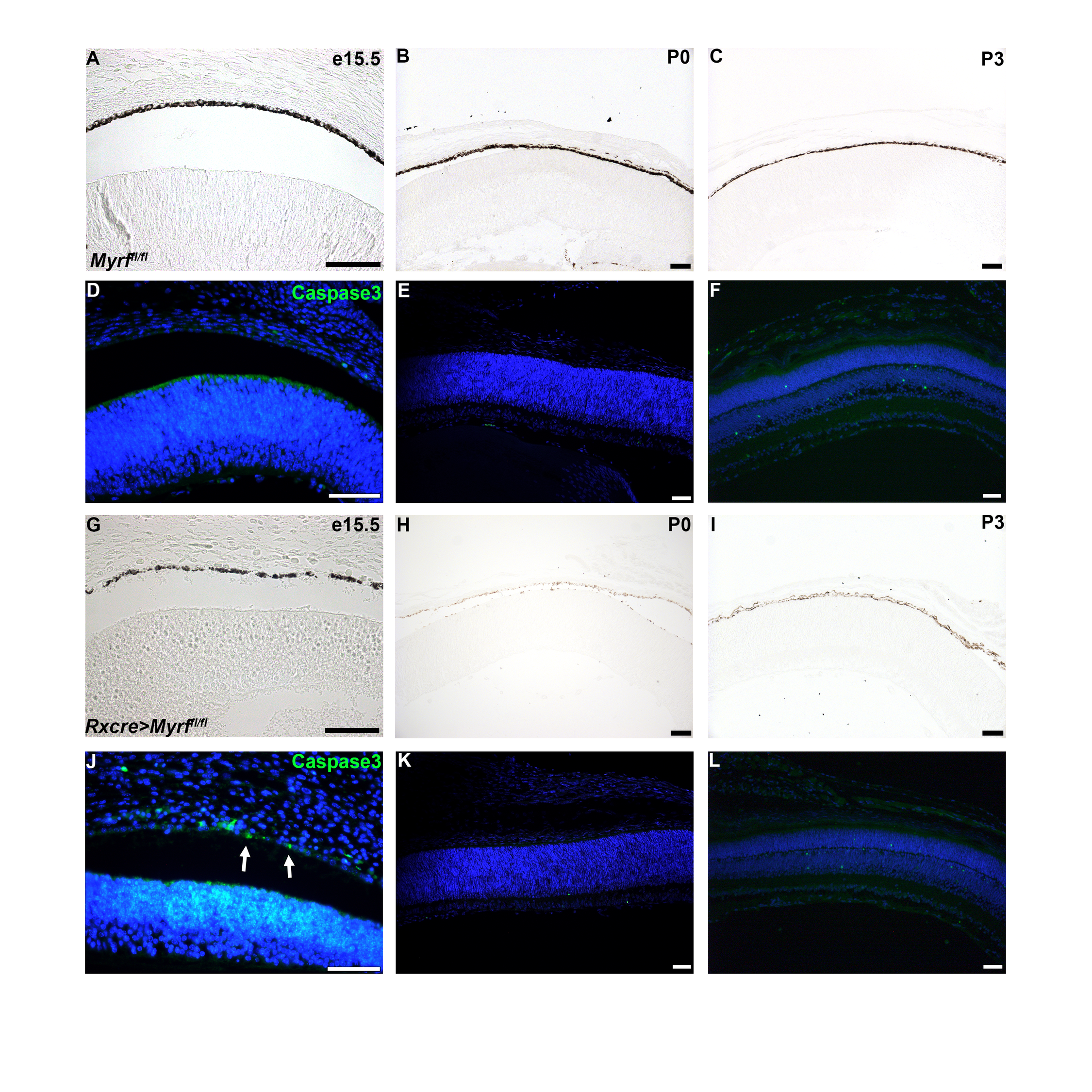

Supplement: S5 Fig — Cleaved Caspase3 immunostaining was used to detect the presence of apoptotic cells in the RPE of Rx > cre Myrffl/fl mutants and controls during late gestation (e15.5) and postnatal timepoints (P0 and P3). Sections through the RPE and retina highlight the pigmentation of the RPE in control mice at e15.5, P0, and P3 (A-C) and the loss of pigmentation beginning at e15.5 in the Rx > cre Myrffl/fl mutants (G-I). Apoptosis was analyzed with cleaved-Caspase3 immunostaining. No apoptotic cells were detected in the RPE of control mice across all timepoints analyzed (D-F). Apoptotic cells were detected at e15.5 in Rx > cre Myrffl/fl mutant RPE, but not in RPE from postnatal mutants (J-L). Scale bars indicate 50um. (TIF) [file pgen.1011670.s005.tif]

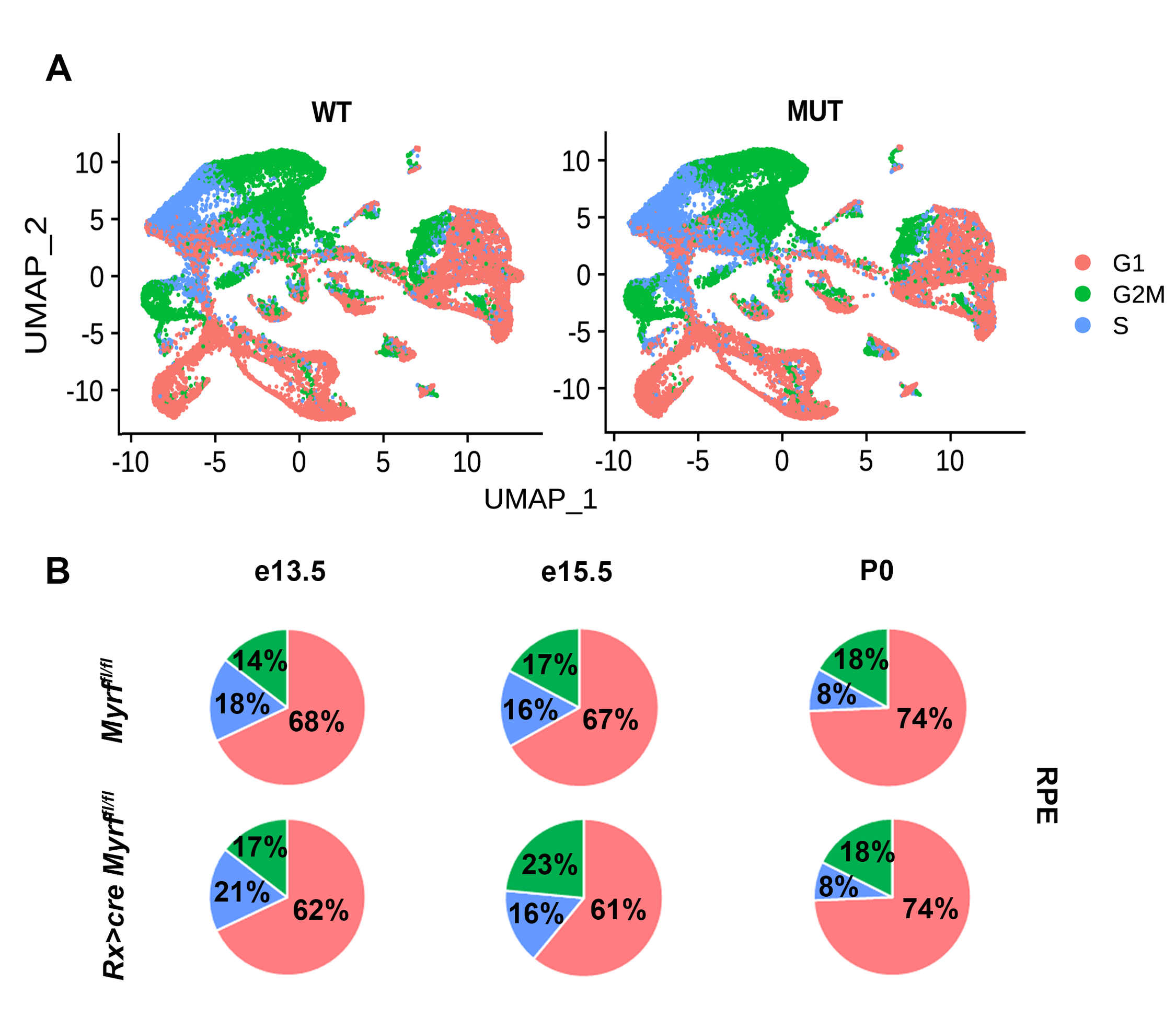

Supplement: S6 Fig — Seurat cell cycle analysis function was used to analyze changes in cell cycle distribution between Myrffl/fl and Rx > cre Myrffl/fl scRNAseq datasets. (A). UMAP FeaturePlot of Cell Cycle Scoring split between WT (Myrffl/fl) and MUT (Rx > cre Myrffl/fl). (B). Pie chart displaying distribution of RPE cells in G1, G2M, and S phases of the cell cycle across each time point and genotype. (TIF) [file pgen.1011670.s006.tif]

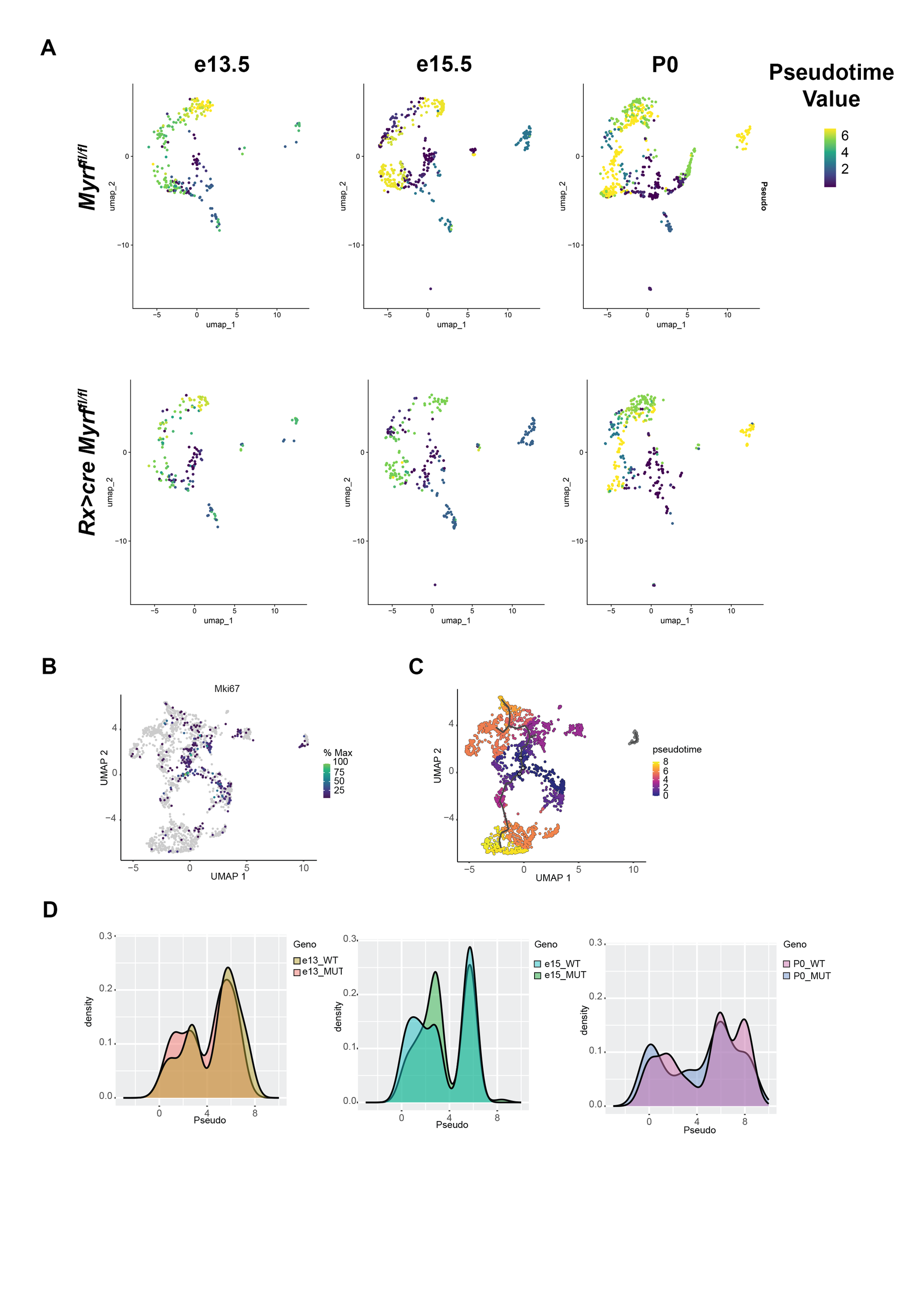

Supplement: S7 Fig — The Monocle3 package in R was used to analyze a pseudotime trajectory across all time points between Myrffl/fl and Rx > cre Myrffl/fl RPE clustered cells. (A) Seurat reclustered RPE UMAP showing distribution of pseudotime values across genotypes. A pseudotime value of 0 indicates a more progenitor like cell state and values increase as the predicted state of differentiation progresses. (B) Expression of Mki67 in the Monocle3 RPE cluster was used to determine the location of proliferating cells to use as the beginning of the pseudotime trajectory. (C)(Pseudotime trajectory in the RPE cluster with point 1 designating the most progenitor like state and points 2 and 3 showing two trajectories of increasing pseudotime values coming from the progenitors. (D) Pseudotime density graph across all genotypes highlighting a modest shift in the Rx > cre Myrffl/fl towards a less differentiated state at P0. (TIF) [file pgen.1011670.s007.tif]

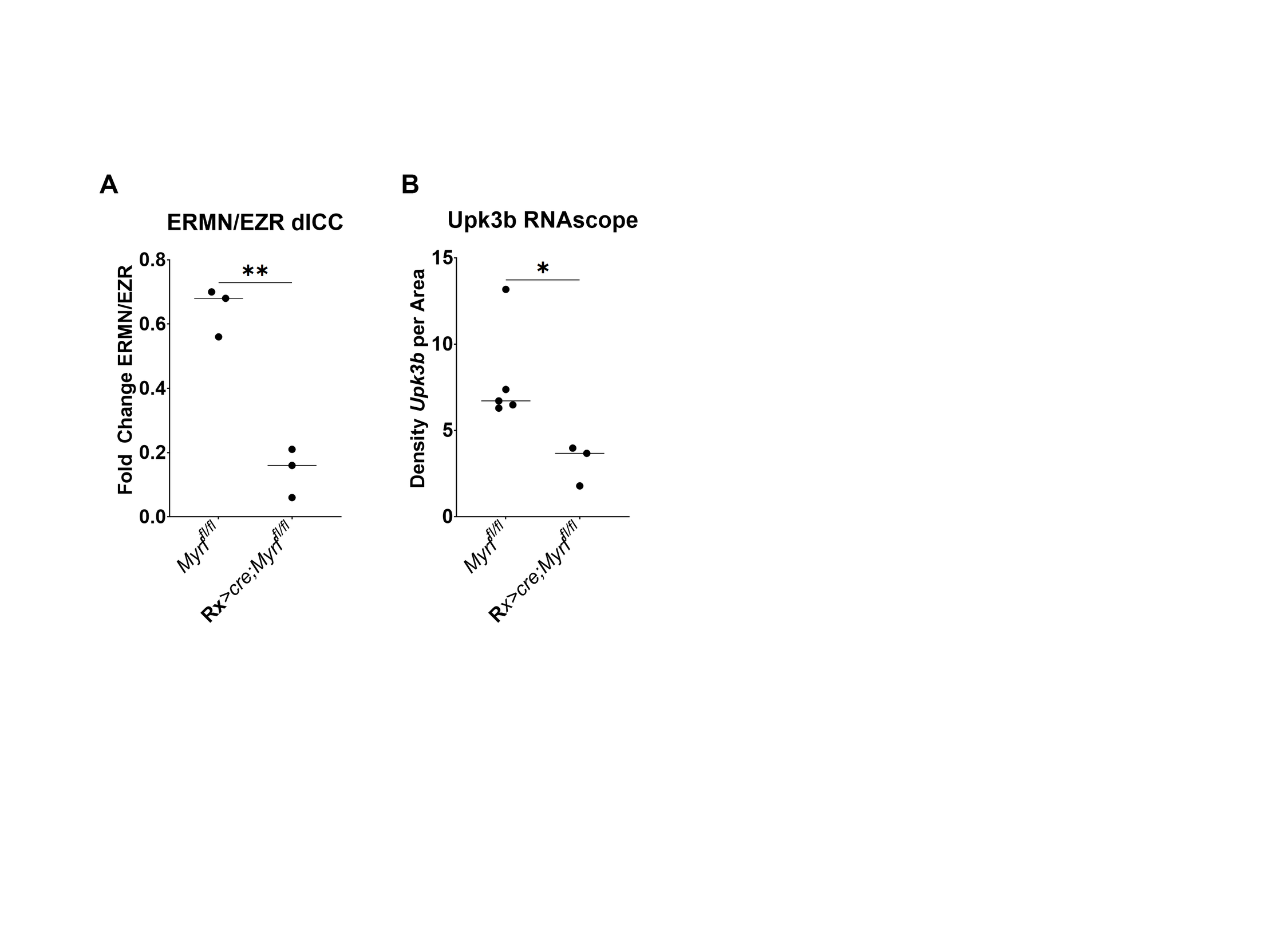

Supplement: S8 Fig — (A) The density of ERMN immunostaining was compared to the density of EZR immunostaining using Image J in both Myrffl/fl controls (n = 3) and Rx > cre Myrffl/fl mutants (n = 3). Statistical analysis was performed using an unpaired T test. ** = p < 0.01, * = p < 0.05. (TIF) [file pgen.1011670.s008.tif]

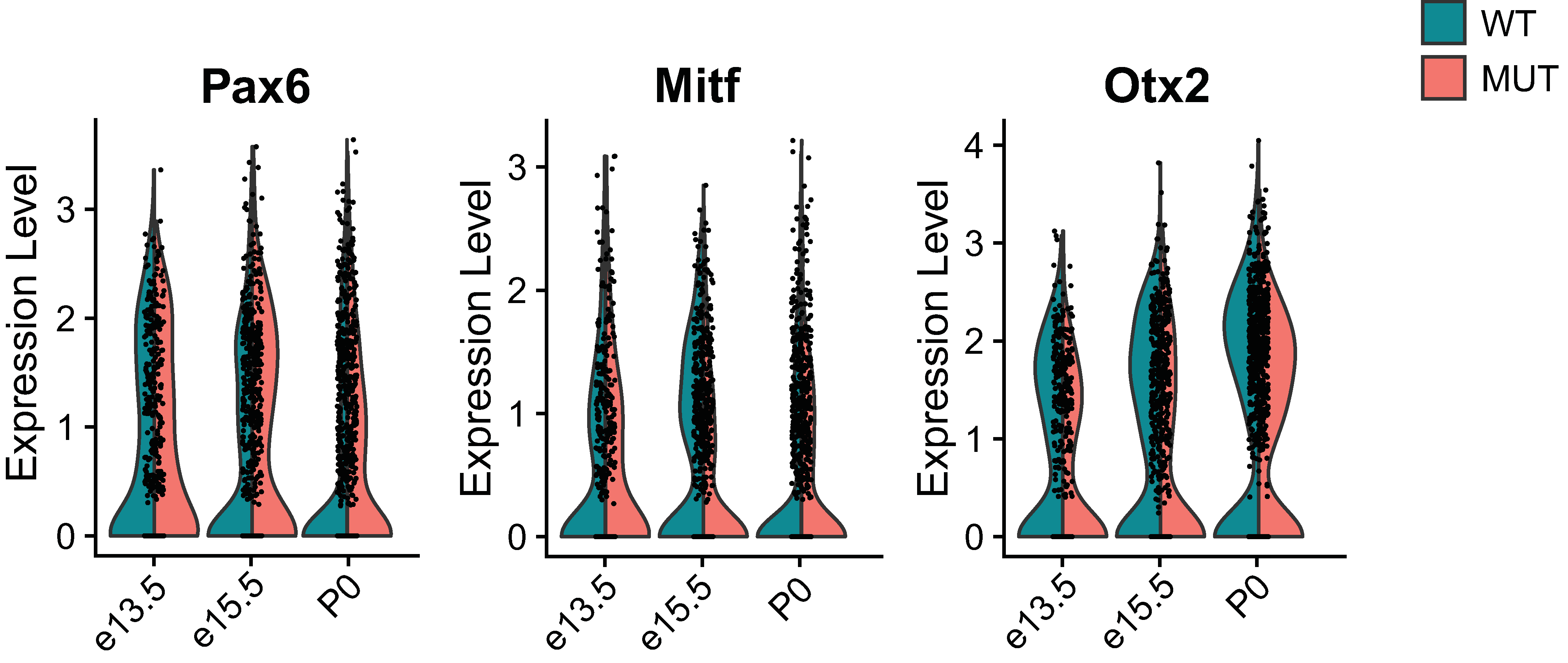

Supplement: S9 Fig — VlnPlot analysis of Pax6, Mitf, and Otx2 in the RPE cluster, shows comparable levels of expression between mutant and wild type across all time points. (TIF) [file pgen.1011670.s009.tif]

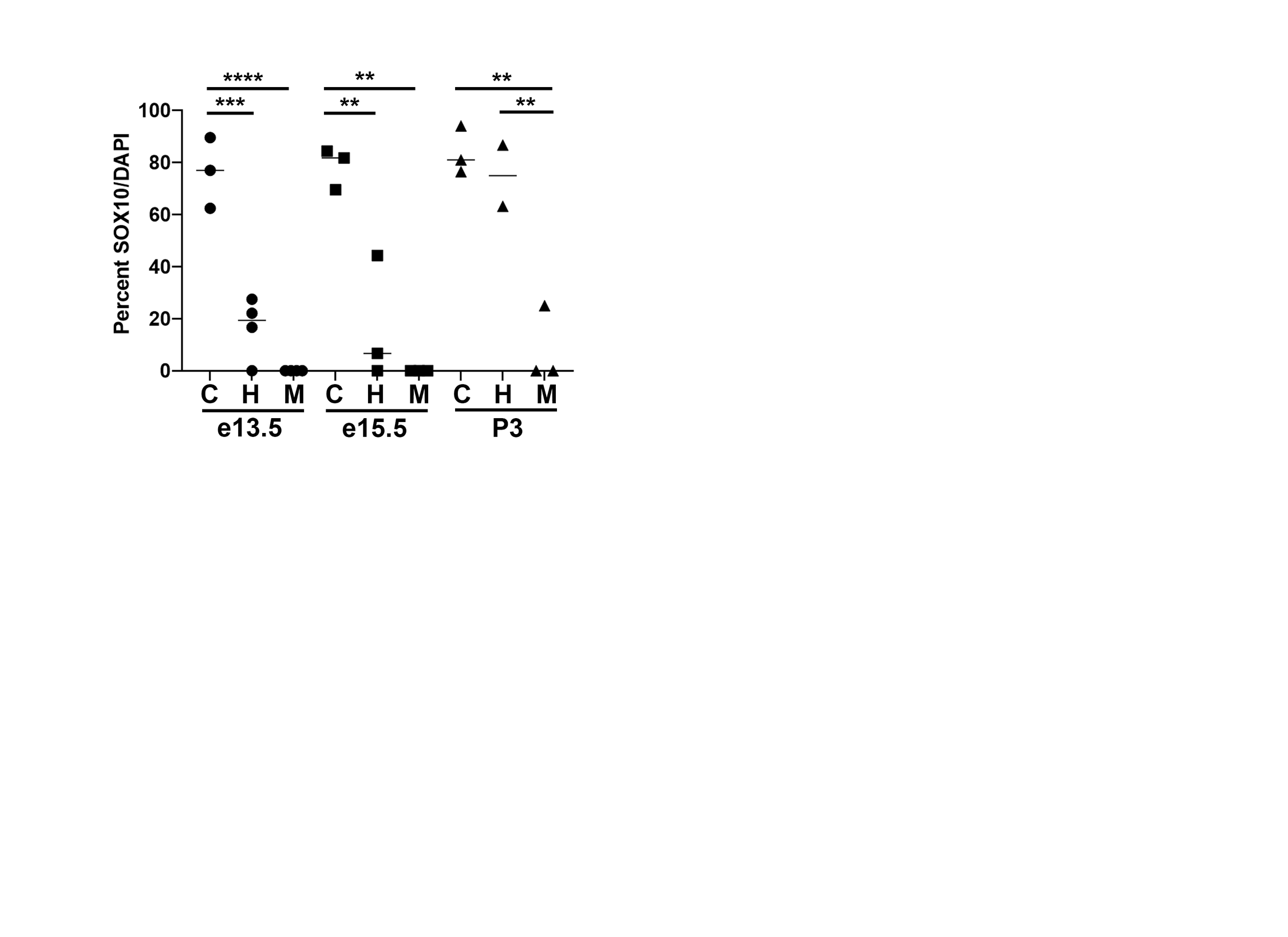

Supplement: S10 Fig — The total number of SOX10 positive cells were compared to the total DAPI cells in the RPE and graphed as a percentage of SOX10/DAPI. Statistically significant differences were seen between the Myrffl/fl (C), Rx > cre Myrf+/fl (H), and Rx > cre Myrffl/fl (M) at all time points. One way ANOVA with Tukey multicomparison was used for statistical analysis. ** = p < 0.01, *** = p < 0.001, **** = p < 0.0001. (TIF) [file pgen.1011670.s010.tif]

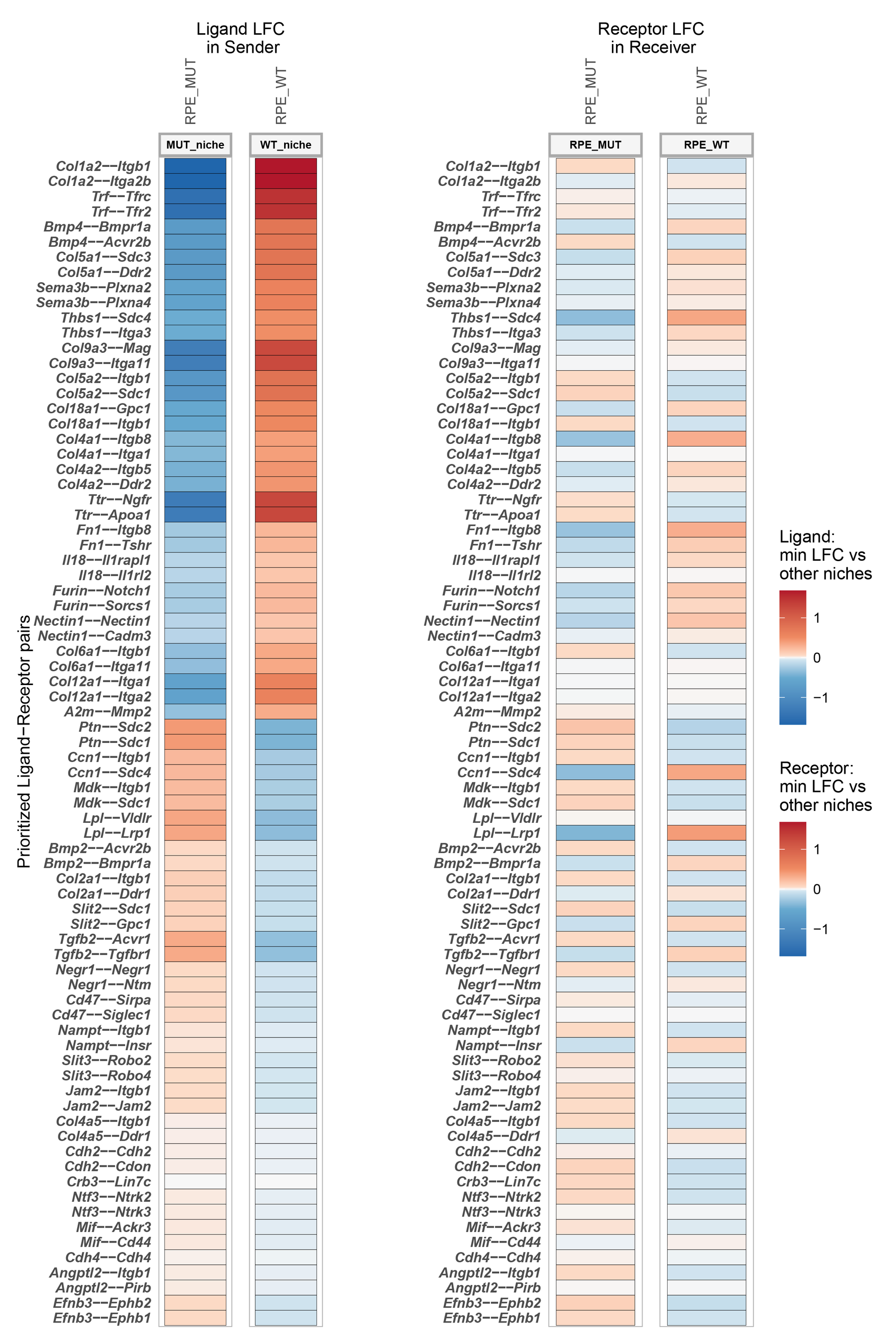

Supplement: S11 Fig — Ligand-receptor pairs from Differential Nichenetr analysis are prioritized based on log fold change (LFC) expression of the ligand in the RPE. Heat maps show expression of the ligand (left) and the receptor (right) within the RPE of the scRNAseq dataset. (TIF) [file pgen.1011670.s011.tif]

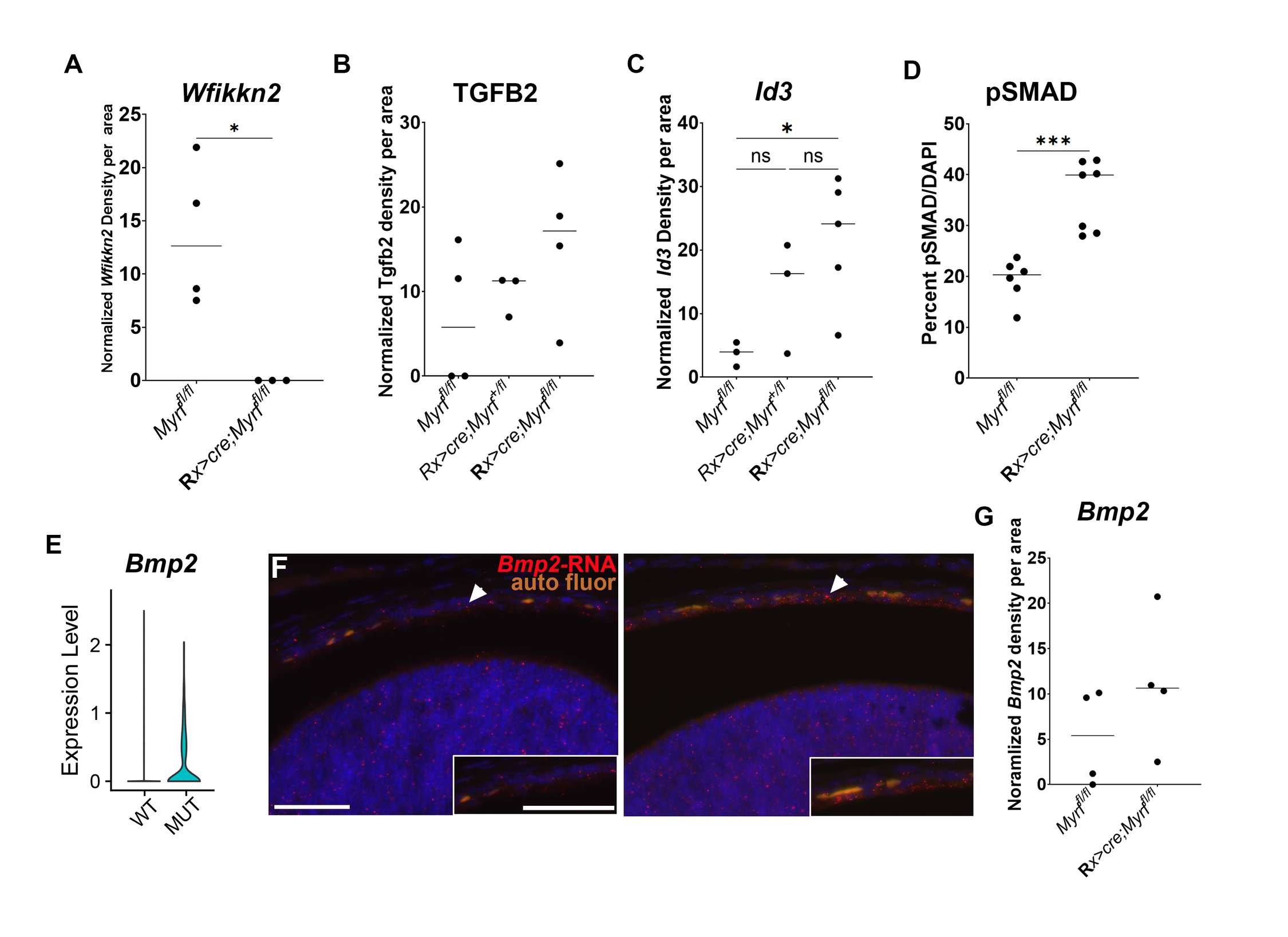

Supplement: S12 Fig — (A) The density of signal per area from Wfikkn2 RNAscope was normalized to background staining in ImageJ. (B) The density of signal per area from TGFB2 immunostaining was normalized by comparing to the density of background staining in ImageJ. (C) The density of signal per area from Id3 RNAscope was normalized by comparing to the density background staining in ImageJ. (D). The density of signal per are of pSMAD immunostaining in the RPE was compared to the density of background staining in ImageJ. (E) VlnPlot of Bmp2 transcript expression in the RPE cluster of the P0 scRNAseq dataset showing a trend towards increased expression in the mutant. (F) RNAscope of Bmp2 demonstrates elevated expression in the RPE of Rx > cre Myrffl/fl mutants (n = 5) compared to Myrffl/fl controls (n = 4). (G). The density of signal per are of Bmp2 RNAscope in the RPE was compared to the density of background staining in ImageJ. Each point on the graphs represents an individual sample. Ordinary one-way ANOVA with Tukey multicomparisons was used to assess statistics in TGFB2 and Id3 staining. The unpaired T test was used to assess statistics in Wfikkn2, Bmp2, and pSMAD staining. * = p < 0.05, *** = p < 0.001. (TIF) [file pgen.1011670.s012.tif]

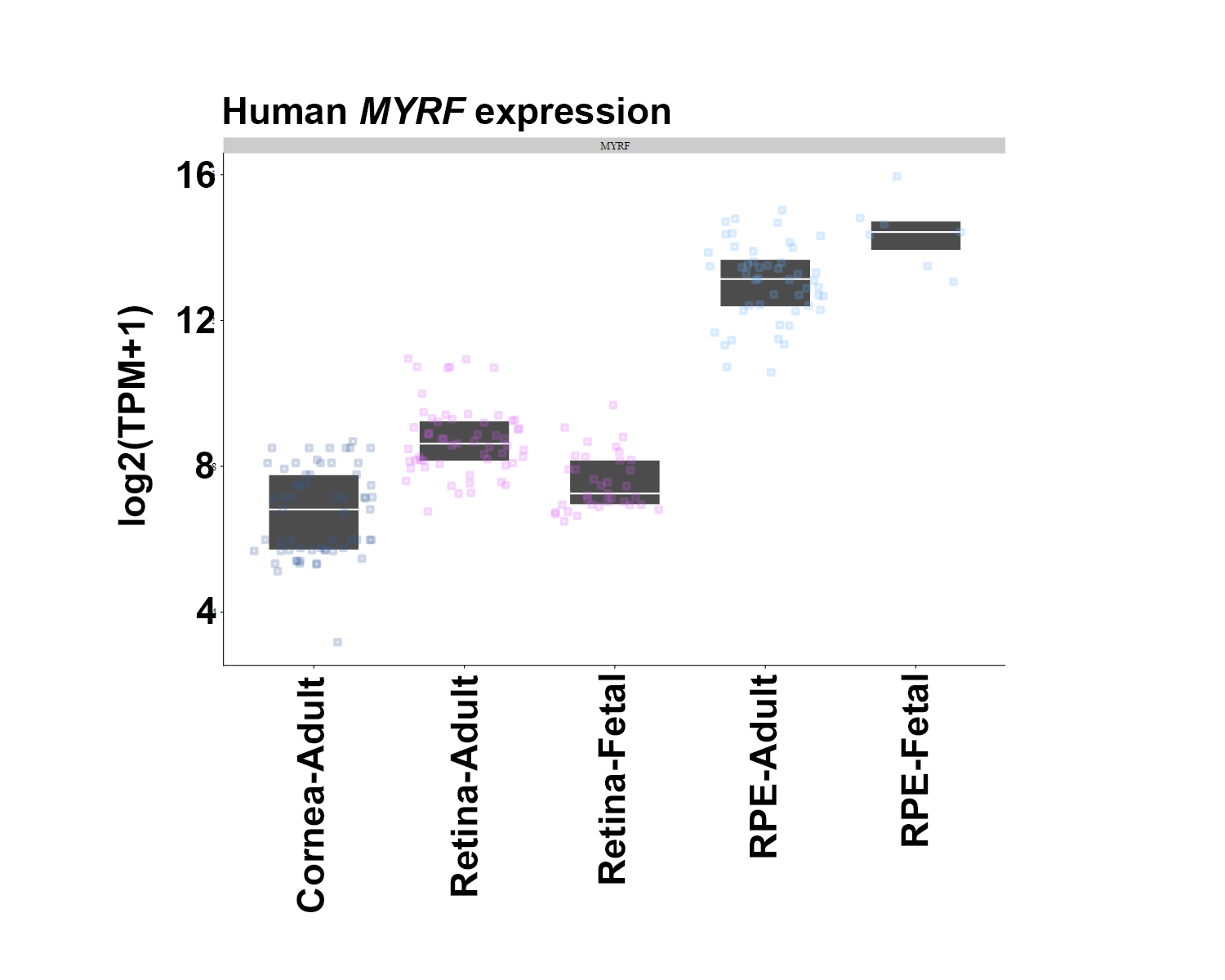

Supplement: S13 Fig — Expression data was assembled from the Eyeintegration website, provided by the National Eye Institute (eyeintegration.nei.nih.gov). A box plot display of the Pan-Human Gene Expression using the Gene 2019 dataset demonstrates exponentially higher levels (at least 4 log fold) of MYRF in the RPE compared to the cornea or retina. (TIF) [file pgen.1011670.s013.tif]

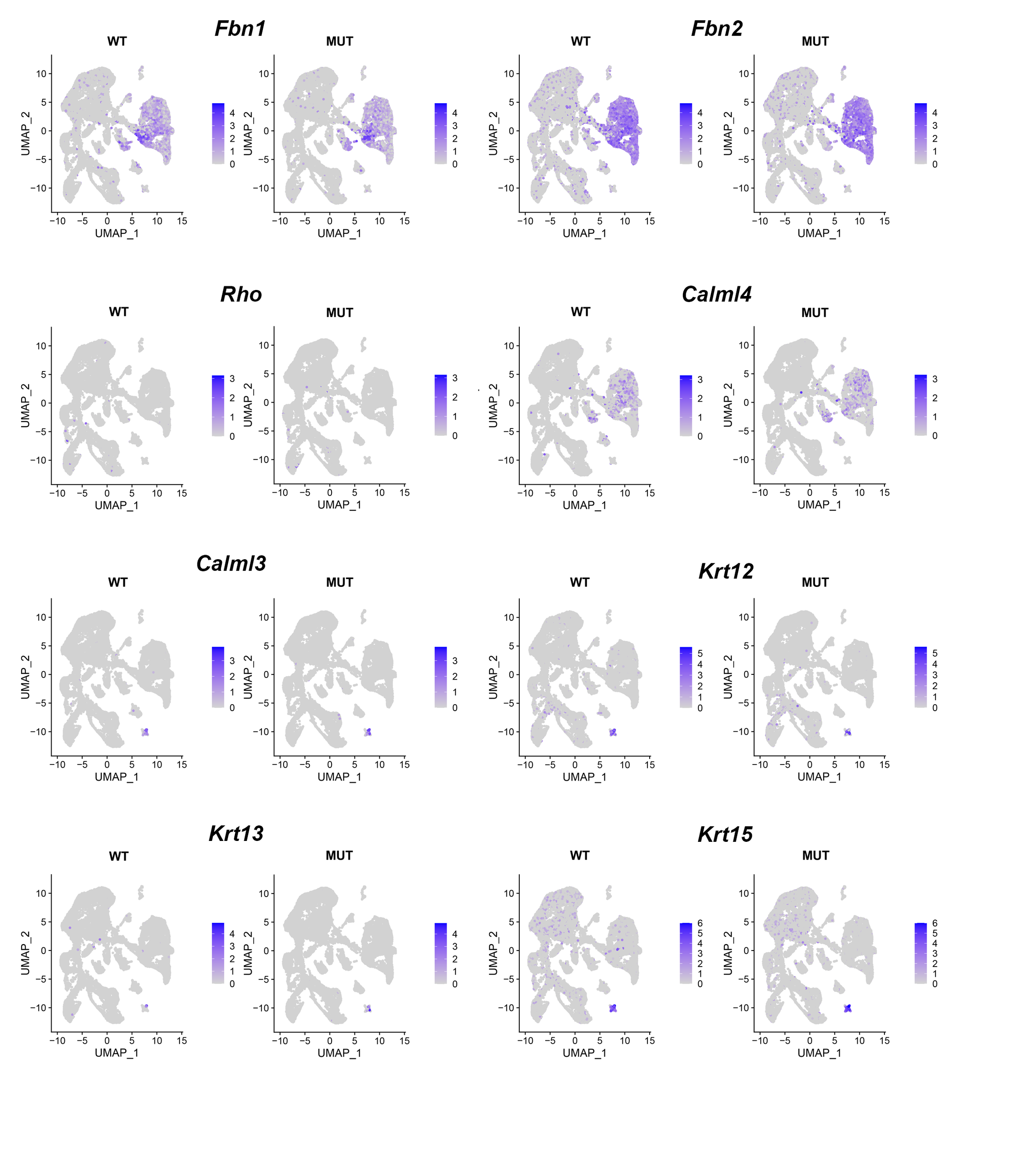

Supplement: S14 Fig — FeaturePlot analysis of genes reported to be altered in other models of loss of Myrf are not altered in Rx > cre Myrffl/fl mutants. (TIF) [file pgen.1011670.s014.tif]
