# Supplementary material for: Myelin regulatory factor (MYRF) is a critical early regulator of retinal pigment epithelial development
Source: PLoS Genet. 2025 Apr 15;21(4):e1011670. doi: 10.1371/journal.pgen.1011670 (PMC12052213; doi:10.1371/journal.pgen.1011670)
Supplement: S1 File — (DOCX) [file pgen.1011670.s021.docx]

**WEB RESOURCES**

Eye Integration: <https://eyeintegration.nei.nih.gov/>

UCSC Genome Browser: https://genomes.ucsc.edu
