# Supplementary material for: Myelin regulatory factor (MYRF) is a critical early regulator of retinal pigment epithelial development
Source: PLoS Genet. 2025 Apr 15;21(4):e1011670. doi: 10.1371/journal.pgen.1011670 (PMC12052213; doi:10.1371/journal.pgen.1011670)
Supplement: Attachment — Submitted filename: Brinkmeier-ReviewerResponse-PLOSGeneticsR1_draft.docx [file pgen.1011670.s023.docx]

We appreciate the reviewers' thoughtful comments and have revised and updated the manuscript to address most of these concerns and provide a point-by-point response below:

*Reviewer #1: The manuscript by Brinkmeier et al performed scRNAseq analysis of conditional MYRF KO mouse eyes and provided a gene expression and some histological analysis to validate a handful of genes in select pathways. Authors show that MYRF is important for RPE melanogenesis, structure, and survival. Lot of these conclusion were published previously by the same lab. This undermines novelty of the current manuscript. Overall, the work is descriptive with frequent speculative conclusions without supporting evidence.*

We respectfully disagree about the novelty of the findings. In the 2019 paper, we showed that loss of MYRF leads to retinal degeneration and a depigmentation of the RPE, and that MYRF interacts with and regulates TMEM98. The current manuscript explores the molecular and physiologic basis underlying these effects. Novel findings include linking Myrf loss to dysregulation of the TGFb-BMP pathways, identification of novel RPE targets, identification of melanogenesis, pigment pathway gene expression as the underlying source of pigmentation phenotype, characterization of the cell death within the RPE, identification of novel targets of *Myrf* including *Ermn* and *Upk3b,* which may be novel disease candidate genes and have very specific expression patterns in the RPE.

Regarding the speculative conclusions, we have modified the text to address these and highlighted the evidence for these in various aspects of the manuscript.

Specific comments:
1) Authors make claims that MYRF is important for RPE function and EMT induction. But no evidence is provided to support these claims.

We agree that the claim for EMT was overstated and have tempered these claims for EMT and instead discussed the support is shown for structural changes in the eye and that there is alteration of gene expression that is supportive for EMT.

2) Authors claim that MYRF is downstream of PAX6 and MITF is not supported by any claim. Authors should analyze MYRF expression in PAX6 and MITF mutant mice.

In the manuscript we argue that MYRF knockout does not affect PAX6 and MITF expression, and it does not affect PAX6 or MITF regulon expression. This is shown in figure 7. Together, these data suggest that PAX6 and MITF are not downstream of MYRF. Additional data supporting that PAX6 and MITF are upstream of MYRF are the phenotypes associated with loss of function. In the case of PAX6 and MITF, global loss leads to microphthalmia or ocular disorganization and disruption of the early eye field. In MYRF knockout, the eye is still formed, and cells differentiate appropriately. *Pax6* and *Mitf* transcripts are not changed between *Rx>cre;Myrf^fl/fl^* and control in our scRNAseq dataset, supporting loss of *Myrf* does not impact early RPE markers.

Assessing the expression of MYRF in PAX6 and MITF mutant mice is beyond the scope of this work. Unfortunately, gene expression data sets from those mutant mice were crude and done at very early timepoints with outdated technologies so are not informative for these questions.

3) The claim that eye development is normal before E14 needs to be confirmed with appropriate evidence.

We have evidence that the eye cup is structural normal, and morphogenesis is complete. The thickness of the retina is normal. We have previously reported that there are no defects in RPE pigmentation prior to e15.5 (PMID: 31048900). We have clarified that morphogenesis appears normal at this age to temper down the conclusions.

4) The claim that SOX10 and MYRF work in the same pathway needs to be substantiated by genetic analysis. Do SOX10 mutant mice phenocopy MYRF mutant mice and the double mutants not have any different phenotype?

While this would be an excellent experiment for the future, genetic analysis of SOX10 mutant mice is beyond the scope of the current work. SOX10 mutants are not available through JAX except with cryorecovery, making analysis of double mutant mice be an infeasible project that would likely take multiple years. We have adjusted our language to focus on what we demonstrate in the manuscript: MYRF regulates SOX10, and that both are involved in similar regulon regulation in RPE. These findings coupled with the body of evidence of SOX10 and MYRF interaction and regulation in oligodendrocytes are suggestive of a shared pathway for regulation, but we have toned down those conclusions.

5) Authors claim that MYRF expression in the RPE is the prime reason for ocular defects in complete null. Without deleting the ON specific MYRF expression, the previous claim is an overstatement. Can authors rule out the possibility that ON-specific MYRF expression does not result is secretion of certain growth factors that regulate RPE and eye development.

Oligodendrocyte myelination and hence MYRF expression in the optic nerve does not begin until well after P0 ( PMID: 22660169). We see the effects of MYRF deletion prior to the initiation of myelination and see loss of pigmentation, RPE findings, and gene expression differences at E13.5, E15.5, and P0, well before myelination initiates. This effectively rules out that optic nerve expression of MYRF is a major contributor to the phenotypes observed and discussed in the manuscript.

6) Clearly MYRF deletion in the RPE result in loss of several hundred genes including critical RPE genes that have been associated with AMD. Without a direct association of MYRF with AMD, this statement is highly speculative. MYRF is a developmental transcription factor and AMD is an age-onset disease.

MYRF is expressed in the adult RPE as well as the developing RPE, thus it has potential to have roles in regulation of both developmental and aging genes. Our statement is meant to specify that MYRF is a regulator of genes important for RPE function, including genes implicated in AMD. We do not make any statement suggesting a causative role.

7) Regulon analysis provided in Figure 8 is interesting by highly speculative and, as authors say, ‘predictive’. It is not a confirmatory proof and needs to be substantiated with additional genetic, epigenomic, and in vitro analysis. No methods are provided for this figure.

Regulon analysis provides a method to evaluate downstream gene expression changes to identify key dysregulated pathways and also support the node of the transcriptional hierarchy and the position of MYRF in the differentiation cascade for RPE. This is using existing biological data so is not speculative, but we agree that it is predictive and not confirmatory. Nonetheless, together with other genetic and functional evidence from the literature, the SCENIC regulon analysis is supportive of our conclusions. Further, we provide validation for key pathways such as the TGF-beta pathway with subsequent immunostaining data. The methods for SCENIC regulon analysis were within the scRNAseq section of Methods. We have created a new Bioinformatic Tools section within the Methods to make it easier to identify.

8) Lines 160-161, how come a 2% reduction in RPE cell number result in in a 8% increase in total RPC number. What is the total number of cells in each case? Are these numbers statistically significant? If yes, are authors suggesting RPE transdifferentiated into retina. If that’s the case, they should provide histological and gene expression evidence in support of that. If not, this data should be removed.

We have shown definitely that RPE is not transdifferentiating into retina. We provide staining with neuronal marker b-III-tubulin, which shows that the RPE does not adopt a neuronal fate in the setting of MYRF loss-of-function, Supplemental Figure 4. Since population differences across all cell populations are inherently difficult to apply statistical tests across different samples, and we do not know about the statistical significance of population reductions, we have removed the suggestion of an increase in the RPC cell cluster. For RPE populations, we have systematically explored cell death and proliferation to evaluate the differences.

9) Lines 177-179, If MYRF is expressed in all of RPE cells, why do only a small number of cells die?

Cell death is a final phenotype followed by cellular dysfunction. It is not uncommon in retinal degenerative conditions that cell death is slow and occurs in not all cells.

10) Lines 185-187 contradict data shown in lines 160-161.

Pseudotime analysis was conducted on the RPE cluster only, while population frequencies were evaluated across multiple time points across the entire cell populations. The results are congruent and not contradictory.

11) Lines 235-237, how do authors conclude depigmentation is due to loss of melanosomes. Can authors rule out slow proliferation of some RPE cells as others are dying? This also doesn’t rule out reduced melanin production. A longitudinal analysis from before the KO and after the KO will help make this claim.

The conclusion is related to counting melanosomes in the electromicrographs. There is a reduction of melanosomes within the RPE and this is noted in the manuscript. Reduced melanin production is possible and melanin biogenesis genes are affected. We have added a statement that this was not ruled out, but our evidence supports that loss of melanosomes is at least a partial contributor.

12) Lines 306-397, why is elevated TGF-beta signaling seen in RPCs? Is this coming from RPE cells and what is the impact of this on RPCs. It seems their proliferation and differentiation is not impacted.

Our Nichenet analysis suggests that RPE signaling to RPCs leads to upregulation of TGFB pathway genes. This is discussed in the results and discussion. Many TGF-beta effectors are secreted molecules and thus have ability to signal to neighboring cells. We discuss these in detail in the manuscript. The effect of upregulation of TGF-beta signaling on the RPC population is not entirely clear – we examined proliferation and differentiation in the context of this study, but further investigation is beyond the scope of the current paper. The relevance to eye growth pathways is an important and interesting finding, and would be a good subject for future studies.

13) Lines 349-350, no evidence is provided for correct RPE specification.

Evidence for correct RPE specification is that the cells form a monolayer, develop some pigmentation, do not express neural markers, and continue to express well established early RPE markers. We updated the text in lines 352-354 to include this.
14) Lines 366-367, no data is provided for loss of RPE hexagonal feature.

We do have data for loss of hexagonal features of RPE in the context of MYRF loss of function, but are including that in a different manuscript so have removed that sentence in this paper. We apologize for this oversight.

15) Lines 398-299, has RPE cell death been reported in MITF KO mice. Don’t these mice have more of a RPE specification defect? Please clarify or correct.

RPE cell death has not be investigated in detail in MITF KO mice, so we cannot comment on this. Our language supports that Mitf is upstream or parallel to Myrf in the RPE differentiation hierarchy for 3 reasons:

1) In human disease and mouse knockouts, the RPE is formed in MYRF LOF, while it is misspecified in MITF. Further, human phenoytpes of microphthalmia or anophthalmia occur with loss of MITF, while there is presence of RPE and eye development in humans with MYRF loss.
2) MITF regulons and MITF expression are not affected in MYRF CKO (suggesting that MYRF is either downstream or acts in parallel to MITF).
3) MYRF CKO RPE, there is normal specification of RPE, but downstream differentiation is affected. Specification defects would by definition be upstream of differentiation defects.

16) What is the evidence and consequence for increased EMT gene expression?

We have tempered conclusions about EMT gene expression and instead discussed these are changes in RPE structure as above in (1).

Reviewer #2: The transcription factor Myrf is critical during eye development in humans; genetic variants can cause nanophthalmos but the underlying functional role for MYRF is very poorly understood. Using wildtype and conditional Myrf mutant mice, this study employed sc RNAseq of mouse optic cups containing retina, RPE and neighboring cell types collected at 3 different time points between E13.5 and birth. One cluster harboring RPE cells together with melanocytes was identified, which was further examined thoroughly with various computational analyses to identify downstream candidates critical for RPE development. This work has great potential to represent an important step toward identifying critical Myrf targets and for elucidating the role of Myrf specifically in RPE and ocular development.

There are 2 major concerns:

1) It appears that the initial clustering yielded a mixed RPE and melanocyte cluster (RPE/Mel), since the identification genes are also expressed in melanocytes (Suppl Fig.1). Is it possible that a rather significant number of cells in this cluster are melanocytes? For example, Fig.2B shows that there are many MYRF-negative cells in the 'RPE' cluster (= RPE/Mel cluster?), however most of the RPE cells in Fig1A (E14.5) and B (P0) are MYRF-positive. The RPE cluster may be able to be separated from melanocytes as a subcluster? For example, Otx2 and Lhx2 are expressed early on and in the postnatal RPE, also shown in Suppl Fig.1. Pax6 is another marker that can distinguish RPE and melanocytes. Also, while the authors define the 'RPE/Mel' cluster in Suppl. Fig.1, they use throughout the manuscript just the term 'RPE' cluster, which is confusing.

We are able to clearly separate RPE from melanocytes and have a separate melanocyte cluster that is apparent on both the UMAP and dot plots. PAX3 was used as a marker to clearly delineate melanocytes as RPE are PAX3 negative, while melanocytes are positive. We have added a supplemental figure showing PAX3 UMAPs to clearly delineate these findings, Supplemental Figure 3. Further, in our SOX10 figure, we are able to see loss of SOX10 in RPE, but see persistence in melanocytes suggesting the specificity of our deletion and the clear clustering of melanocytes and RPE separately. The RPE/Mel label at the top of the dotplot in Supplemental Figure 1 was meant to guide the reader across the dotplot showing which markers are expressed in which clusters. This label marks genes shared by the two clusters and was not meant to suggest the identification of a mixed cluster. We understand how this can be confusing to the reader and have removed the top labels from the dotplot.

2) In many figures, colocalization with relevant genes in RPE or other structures such as optic nerve, melanocytes or appropriate higher magnifications are missing, among them Fig.1, Fig.6 (incl. a previously unknown gene expressed in RPE UpK3b), Fig.7. This applies particularly to Fig.9, the magnification is too low to confirm whether the labeling of TGF pathway genes is present in RPE cells. This is important for validation of the scRNAseq data, especially if this a mixed RPE/melanocyte cluster.

Insets have been added to Figures 1, 6, and 9 to more clearly delineate labeling.

There is also a concern that very few cells in the RPE in Myrf mutants show upregulated pSMAD labeling, and the effect on TGFB2 and BMP2 is not very clear.

We have used multiple methods for validation of the increase in TGFB/BMP signaling. We have looked at a variety of techniques including scRNAseq, IHC, and RNAscope along with looking at multiple components of the pathway. For each experiment we have looked at 3-6 samples of each genotype. Taken together, this is suggestive of an upregulation of this signaling pathway.

And what does the green labeling in Fig.9E in wildtype tissue represent?

The green cells in Figure 9E represent autofluorscent blood cells, this has been added to the figure and figure legend.

Fig.7: Sox10 expression appears to variable at E13.5, compared to expression in melanocytes, and very sparse at P0. Also here, it would be helpful to see colocalization with RPE marker.

The expression level level in the RPE is lower compared to melanocytes which makes it appear as variable, but it is present in all the cells of the monolayer.

Minor points
- Rx-Cre can also be expressed in the lens. Please, provide information whether MYRF is expressed in lens.

Our single cell data had the lens removed, but in our RNAscope in situ of MYRF, we do not see expression of MYRF in the lens. We added a panel to Figure 1 and updated the text.

- Is there anything known about Sox10 function in the developing eye?

SOX10 is involved in Waardenburg syndrome in patients, which may have similar features to ocular albinism with blonde fundus, foveal hypoplasia, iris transillumination defects. The role of Sox10 in the mouse eye is not well-studied, so we cannot comment on its function and that would be an interesting area of future work.

- How is "highest intensity Otx2 stained" or 'less intensity stained' defined?

This designation was meant to distinguish between photoreceptor (high) and retinal progenitor derived Otx2 (low). However, we agree that high vs. low intensity Otx2 staining does not have a systematic cutoff, so we have omitted this and focus on overall Otx2 expression in the figure.

Reviewer #3: In this study, Brinkmeirer, Prasov and colleagues investigate the role of MYRF in RPE cells using conditional deletion in mice and employing single cell RNAseq analysis. The data presented, as well as the analysis is of high quality and well controlled. I do feel the study in its current form falls a bit short of PlosGen standards, though. Some follow-up experiments could add the needed depth. The authors go to great length arguing an RPE-centric roll for Myrf function on eye size control and PhR health. But the critical experiment of deleting myrf only within RPE is missing. Whole optic cup deletion with rx:cre leaves the window open for other interpretations. Even though myrf is more highly expressed in the RPE as compared to other parts of the eye, the protein could still have supplementary and significant roles outside of the RPE.

We have included an updated experiment using RPE-Tyr-CreERT2 to delete Myrf early in development and specifically in only RPE, Supplemental Figure 1. This experiment confirms that pigmentation changes and loss of TMEM98 expression are present and phenotypic appearance is similar. Together with our expression data suggesting that MYRF is most highly expressed in RPE, our results support that effects that we are seeing in Rx>Cre MYRF floxed mice are the result of RPE loss of MYRF.

Some follow-up experimentation with the other major conclusions is also warranted. First, Sox10 is implied as a direct target of Myrf. This should be evaluated.

We generally show support that Sox10 is a target of Myrf, based on regulon changes as well as early loss of Sox10 in Myrf CKO mice. We cannot definitively conclude that Sox10 is a direct target of Myrf, which would require CUT&RUN or CUT&TAG experiments. Those are challenging experiments given the lack of a specific antibody for MYRF that can be used in the mouse eye, and while they may be the topic of future work, are currently not feasible in the context and scope of this study.

Last, it is observed that BMP/TGFb signaling is enhanced in myrf cKO eyes. What is the basic mechanism underlying this effect? Any does this pathway activation drive subsequent phenotypes. Yes, literature supports this possibility, but I think experimental tests should be conducted.

Our suggested mechanisms for the upregulation of BMP/TGFb signaling in CKO mice is that there is downregulation of inhibitors of BMP/TGFb pathway components (i.e. loss of Wfikkn2) and upregulation of activators of this pathway (i.e. upregulation of Bmp2) in the context of Myrf CKO. We show that these in turn are supported by MultiNicheNet analysis suggesting ligand-receptor interactions within the RPE and between RPE and neighboring cell types contribute to these results. Additional knockout or knockdown experiments may be a topic of future evaluation but are beyond the scope of the current work.

In short, this is a very solid start to a potentially compelling study. My recommendation is the push the experimentation just a little further to really enhance the impact of this nice research.

We thank the reviewers for these thoughtful comments.
