# Supplementary material for: Myelin regulatory factor (MYRF) is a critical early regulator of retinal pigment epithelial development
Source: PLoS Genet. 2025 Apr 15;21(4):e1011670. doi: 10.1371/journal.pgen.1011670 (PMC12052213; doi:10.1371/journal.pgen.1011670)
Supplement: Attachment — Submitted filename: BrinkmeierPLoSGenetics2025-Myrfsc-R2-response-final.docx [file pgen.1011670.s024.docx]

We have revised the manuscript according to the reviewer and journal comments and provide a point-by-point response below.

Reviewer's Responses to Questions

Reviewer #1: none

We appreciate that our revisions have satisfied the concerns of reviewer #1.

Reviewer #2: The revisions do not address satisfactorily many of the concerns.

In the revised version, the authors provided higher magnifications of the presumed RPE region in insets in several figures. However, this does not resolve my concern whether gene expression is localized to RPE. One way to address this is trying RNAscope double-labeling with 2 different probes or combining RNAscope with immunohistochemistry. Also, if double-labeling with RPE markers is not possible with the validated Myrf antibody that the authors used in their previous study (Garnai et al., 2019), the current Myrf transcript labeling should be at least overlaid with brightfield at high magnification to show overlap with RPE pigmentation in controls and also in Myrf CKO, since some pigmentation is still present.

We have provided additional data in the manuscript to support RPE specific expression of MYRF and highlight some of the existing data in the manuscript that supports this. First, in the scRNA seq data (E13.5-P0), Myrf expression is only evident in the RPE cluster and not in other clusters, and this expression is decreased in the conditional knockout of Myrf. Second, pseudobulk analysis in supplemental table 4, support that Myrf is expressed only in the RPE cluster and not melanocytes or other surrounding ocular structures and its expression is lost in the knockout. Third, we include the autofluorescence channel which was captured along with our images, revised Figure 1. In these images, high level autofluorescence occurs from red blood cells within the choroid, which is positioned behind the RPE. Red blood cells are known to autofluoresce in embryos and are easily defined by their donut and smooth morphology (PMCID: PMC5657453). The RPE forms a single epithelial sheet and is a clear monolayer as evidenced by OTX2 staining in Figure 3. Based on its location behind the neural retina and in front of the choroidal red blood cells, the RPE monolayer of cells readily identified by its location and morphology. Further, in addition to immunofluorescence staining or RNAscope in situ hybridization, we provide evidence of RPE specific expression of genes such as *Upk3b* and *Ermn* in our scRNAseq UMAP Feature plots, dotplot and violin plot analysis.

Also, were the images processed the same way?

Images were processed in the same way with same exposure.
Instead of doing double labeling we can definitively identify the RPE using autofluorescence channel that marks the underlying choroidal red blood cells.

The magnification in Fig.1K is too low, and the orientation in L is unclear.

We have provided a better image of the ciliary body and have labeled the panel for better orientation to address this comment

Is the lens in Fig.1L shown as a whole?

We have provided a better image of the lens and labeled the panel for better orientation, so that the lens is clear.

Overall, with these corrections, this figure would then also more convincingly confirm their previous study from 2019.

Figure 1 goes beyond the prior study in 2019, by confirming the distribution of Myrf in the mouse RPE, as this was only evaluated in human RPE in the 2019 paper. Further we provide a developmental time course of Myrf expression, which has not been previously reported. These both are particularly important given recent discrepancies about the site of action of Myrf within the eye (with suggestions that RGCs and ciliary body may play a role in Myrf phenotypes). However, we debunk these experiments done with flawed antibody reagents and definitely show that the RPE is the primary site of expression of Myrf throughout development. Our 2019 study confirmed the poor specificity of an MYRF antibody, and we have further tried multiple different other reagents that do not lose staining signal in the conditional knockout (suggesting poor specificity for MYRF antigen).

Similarly, in Fig. 6, RPE expression of Upk3b would be novel, however this needs to be shown with an RPE marker colocalized.

The RPE is readily identified in retinal histologic sections as it forms a monolayer behind the retina and in front of the choroid as noted above. We have included the autofluorescence channel, which shows choroidal RBCs to better delineate the location of the choroid. The outlines of the retina are readily apparent given the tightly packed nuclei in the retinal layers. As such, complex double in situ or in situ/immunofluorescence staining experiments are not required to confidently identify the RPE monolayer in our sections/images. Further, specificity of marker expression for Upk3b in RPE, is supported in our scRNA where this is the only cluster with high level expression. Pseudobulk data are also provided in supplemental table 4.

In Fig.7, it appears that Sox10 is only weakly expressed in conditional hets at E13.5?

We quantitated the number of SOX10 positive cells in the RPE per the number of DAPI stained cells to show a reduction in SOX10 in the conditional mutants. There was not a statistically significant reduction in SOX10 expression in the conditional hets. We have included Supplemental Figure 10 and Data Table 1 to highlight the quantitative analysis.

In general, changes in RPE development can affect differentiation of extraocular tissues, thus, it should be confirmed that Sox10 is expressed in melanocytes.

Our focus on Sox10 expression is in the context of autonomous effects within the RPE. However, we have added pseudobulk RNAseq analysis, supplemental table 4, confirms the expression of *Pax3* and *Sox10* in the melanocyte cluster and that this expression is unchanged in Myrf conditional knockout mice. Within our single cell data, Sox10 is detected only in the melanocyte and RPE clusters and expression is unchanged in the melanocyte cluster, but decreased in RPE consistent with the immunostaining results.

In Fig.9, the effects on TGFb2, BMP2, Id3 and pSmad expression in CKO RPE are not convincingly shown, whether it is scRNAseq data (Fig.9A) or RNAscope or IHC for pSMAD (C-F). This need to be quantified and colocalization with RPE markers at higher magnification needs to be shown. Overall, the key conclusions for this study are not supported by the data presented here.

We have revised our Figure 8 and Figure 9 of the TGFb/BMP signaling. We have moved the DotPlot of all the TGFb/BMP signaling family members to Figure 9A and have included a VlnPlot at P0 of the pathway members we follow up on with significant changes, Figure 9B. We have also quantitated the density of staining for *Wfikkn2,* TGFB2, *Bmp2,* *Id3*, pSMAD to assess differences between controls and mutants. We have included Supplemental Figure 12 and Data Table 1.

Comments regarding new data:
Fig. S1: The data obtained for disrupting Myrf in the developing RPE using Tyrp-Cre-ERT2 is not convincing. The occurrence of Myrf transcript (red label) in the region of the RPE in the CKO does not really look different from control. Quantification should be provided.

We have looked at pigmentation, Tmem98 staining, and Myrf RNAscope across 3 independent RPE-TyrCreER mice. In each case, we see decreased pigmentation, Tmem98 staining, and Myrf reactivity, though given the inefficiency of the Cre the effects can be regional in areas where Myrf is not fully deleted. We have quantified the fluorescence for Tmem98 and Myrf RNAscope across these animals and find that it is significantly different.

And where would the choroid in all samples be located?

Again, we have included the autofluorescence channel to easily identify the choroid, as choroidal red blood cells autofluoresce and are readily recognized by their smooth donut shaped morphology. This helps us definitively localize the RPE.

How many animals per genotype were analyzed? Labels for tissue types, lines and arrows would be very helpful.

We have included N=3 animals, and included labels for tissue layers to make this easier to read.

Additional comments:
The manuscript needs thorough checking whether revisions have been completed for every section (including methods), and whether subtitles need to be adjusted. For example, the subtitle in line 144 is not accurate anymore, since only one population (RPE) appears altered in Myrf CKO.

We changed this subtitle to loss of MYRF alters RPE cell number during development. We have thoroughly checked the figure and supplemental figure numbers to ensure proper citation throughout the manuscript and can fix any discrepancies if they arise.

Fig.3: TUNEL
The units for the y-axes should be added. T-test is not appropriate if 3 groups are compared. It should be mentioned whether both eyes for each animal were analyzed.

We agree that ANOVA is the appropriate test along with Tukey’s for multiple comparisons and we have modified the statistical testing for Figure 3 as per this comment. We have changed the units to % and used an Ordinary one-way ANOVA with Tukey multicomparisons for our statistical analysis. In most cases, both eyes for each animal were analyzed.

Line 82: The word "controls" is in the wrong place.

This is referring to line 177 Rx>Cre Myrf fl/+ instead of Rx>Cre Myrf fl/fl and has been fixed.

Overall, the scRNAseq data is described in a fine way and points to potentially interesting mechanisms of Myrf's role during RPE differentiation. It would be significantly enhanced, if causative relationships between Myrf and downstream factors (Sox10, TGFB/BMP) are mechanistically investigated.

We thank the reviewer for their thoughtful comments regarding the scRNAseq data. Additional knockout and overexpression studies are topics of future direction, but currently out of the scope of the study at hand. We appreciate these caveats and have been careful in the manuscript text to not overstate our conclusions. Our manuscripts in current forms contains 10 figures and 14 supplemental figures, provides bioinformatic data, histologic confirmation, and validation of our transcriptomic analysis. Our work support an important role of Myrf in RPE differentiation and provide a significant advance in the field even without the further requested genetic experiments, which would take multiple additional years to complete.

Reviewer #3: While additional analysis would strengthen this work, my main concern about RPE autonomy was addressed.

We thank the reviewer for their comments and some of the work is the subject of current and future directions in the laboratory but outside of the scope of the current manuscript.
